# Isolating subsurface fluorescence in macroscopic lifetime imaging via high-spatial-frequency structured illumination

**DOI:** 10.1088/2515-7647/ae0aa1

**Published:** 2025-10-06

**Authors:** Nanxue Yuan, Saif Ragab, Navid Nizam, Vikas Pandey, Amit Verma, Tynan Young, John Williams, Margarida Barroso, Xavier Intes

**Affiliations:** 1 Center for modeling, simulation and Imaging in Medicine, Rensselaer Polytechnic Institute, Troy, NY 12180, United States of America; 2 Department of Molecular and Cellular Physiology, Albany Medical College, Albany, NY 12208, United States of America; 3 City of Hope, Beckman Research Institute, Department of Molecular Medicine, Duarte, CA 91016, United States of America; 4 Department of Biomedical Engineering, West Chester University, West Chester, PA 19380, United States of America

**Keywords:** FLI, FRET, MFLI, HSF, structured light illumination, MCX

## Abstract

Macroscopic fluorescence lifetime imaging (MFLI) has emerged as a robust, non-invasive imaging technique offering quantitative insights into physiological and molecular processes within live tissues, independent of fluorophore concentration, excitation intensity, or signal attenuation. However, a key limitation is the inability to accurately determine the depth at which fluorescence signals originate, potentially compromising biological interpretation due to ambiguous localization. In this study, we introduce high spatial frequency-fluorescence lifetime imaging (HSF-FLI), an innovative optical correction methodology designed to effectively eliminate surface signal bias, such as those arising from skin in preclinical imaging, without requiring chemical clearing agents. We develop a modulation transfer function linking spatial frequency with signal penetration depth through comprehensive Monte Carlo eXtreme simulations. Utilizing structured, three-phase sinusoidal illumination, fluorescence signals were accurately decomposed into distinct surface and subsurface components. Experimental validation was performed using agar-based capillary phantoms and a time-gated intensified charged coupled device coupled with a digital micromirror device imaging system. Further demonstrating its practical utility, we successfully applied HSF-FLI to preclinical drug delivery assessments employing Förster resonance energy transfer MFLI. The method was rigorously validated *in vivo* using mouse tumor xenograft models and cross-validated through *ex vivo* analyses. Overall, by integrating structure illumination techniques with physics-based depth modeling, HSF-FLI achieves precise depth-selective FLI. This advancement significantly enhances the accuracy, biological interpretation, and applicability of FLI, positioning HSF-FLI as a valuable tool for translational research.

## 1 Introduction

Fluorescence lifetime imaging (FLI) has emerged as a transformative tool in molecular biology, uniquely enabling spatially resolved, quantitative measurements of key biochemical and biophysical parameters, including pH, ion concentration, oxygen tension, redox state, and molecular interactions [1–4]. FLI quantifies the fluorescence decay time of fluorophores following excitation [5, 6]. A key strength of this technique is that fluorescence lifetimes are intrinsic properties of fluorophores and their molecular environment, largely independent of fluorophore concentration, excitation intensity, and tissue signal attenuation [1, 7–10]. As a result, near infrared (NIR) FLI has gained significant traction in macroscopic imaging of biological tissues (MFLI). MFLI is used in preclinical *in vivo* imaging of small animal models to study pharmacokinetics, drug delivery, immune responses, and the tumor microenvironment. Its utility is also expanding into clinical domains, including fluorescence-guided surgery and ophthalmic diagnostics [11–13]. A particularly powerful application of FLI is its ability to quantify Förster resonance energy transfer (FRET), a distance-dependent energy transfer between donor and acceptor fluorophores that reports molecular interactions at $ < $10 nm resolution [11, 14–17]. By monitoring the reduction in donor fluorescence lifetime, FLI-FRET enables the quantitative assessment of protein–protein interactions and conformational changes in live, intact biological tissues [18–20]. This capability also allows for the quantification of antibody–target engagement within native tissue environments, an essential parameter for evaluating the efficacy of targeted therapeutics [21–23].

At the macroscopic scale, light propagation in biological tissues is predominantly governed by the scattering properties of the sample. As a result, fluorescence signals are collected from extended three-dimensional regions, making it challenging to resolve the depth of fluorescence signal origin without more sophisticated approaches, such as fluorescence LiDAR [24–28] or full 3D fluorescence tomography [29–32]. In the commonly used wide-field imaging configuration, each pixel can contain a mixture of signals originating from various depths, potentially introducing significant bias in biological interpretation [33–35]. To address scattering effects, optical clearing agents have been developed to homogenize refractive indices within tissues, thereby improving light penetration [36, 37]. However, these agents are known to quench fluorescence signals [38, 39] and may denature tissue structures *in vivo* [40–42], altering the microenvironment of the fluorophores. These alterations can compromise fluorescence lifetime measurements and introduce uncertainty in the quantification of targeted drug delivery. An alternative strategy involves using structured illumination with sinusoidal excitation patterns to modulate the light penetration depth, enabling optical sectioning to separate in-plane from out-of-plane fluorescence signals [43, 44]. While promising, current structured illumination models are limited in their ability to determine the depth correlation of spatial frequency and have only been applied to intensity-based imaging [45, 46]. Structured illumination has also been explored in the microscopic regime for FLI, where optical sectioning was demonstrated at cellular scales [47, 48]. However, these applications were restricted to thin, low-scattering samples with fields of view on the order of millimeters, where photon propagation is largely ballistic [49] and depth penetration is inherently shallow. In contrast, extending structured-illumination lifetime imaging into thick, highly scattering tissues remains a critical gap. In such environments, multiple scattering events randomize photon trajectories [50], severely obscuring subsurface signals [46] and preventing reliable lifetime quantification. This limitation is particularly problematic for *in vivo* applications, where the ability to resolve drug–target engagement within intact, heterogeneous tissues is essential yet remains largely unaddressed.

To overcome these limitations, we propose a high-spatial-frequency FLI (HSF-FLI) framework that enhances lifetime accuracy by selectively suppressing surface-scattered signals. Instead of performing full tomographic reconstruction, our method achieves a practical separation of fluorescence into superficial (skin or surface) and subsurface (deeper tissue) components. This distinction is crucial for *in vivo* imaging, where surface signals often dominate and bias biological interpretation. We implemented HSF-FLI on an MFLI system equipped with structured illumination, derived a depth-correlated modulation transfer function (MTF) via Monte Carlo (MC) simulations and validated the approach in phantoms and mouse tumor xenografts. By removing superficial contamination, HSF-FLI provides a robust and non-invasive way to improve the accuracy of subsurface lifetime measurements in preclinical studies.

## 2 Methods

### 2.1 MFLI intensified charged coupled device (ICCD) imaging system

#### 2.1.1 MFLI ICCD setup

The MFLI system is equipped with a time-gated ICCD (Picostar HR, LaVision, GmbH, Bielefeld, Germany) [24]. HSF-FLI data was acquired for 101 gates with 300 ps gate width and 80 ps gate step (8 ns of overall acquisition time). An 80 MHz tunable Ti: sapphire laser (Mai Tai HP, Spectra-Physics, CA,60 USA) with a laser period of 12.5 ns provided excitation in the NIR range (690 nm–1020 nm). The laser beam was directed into a digital micromirror device (DMD), (DLi 4110, Texas Instruments, TX, USA) through a multi-mode optical fiber. The DMD enables the projection of controlled spatial patterns encoded over 255 levels of gray. The DMD was controlled by a customized Labview script, which projects three pre-made 8-bit sinusoidal patterns with an offset of 2*π*/3 for each selected spatial frequency. Fluorescence-emission filters and neutral density filters were used for FLI and instrument response function (IRF) capturing, respectively. The IRF was captured using white paper.

#### 2.1.2 Imaging Process

To perform fluorescence imaging from the MFLI system, stabilized pulsed illumination was used (Mai Tai HP, Spectra-Physics, CA,60 USA). Imaging was performed using the ICCD camera (Picostar HR, LaVision, GmbH, Bielefeld, Germany) with a gate width of 80 ps and 101 gates were acquired for complete fluorescence decay acquisition. The IRF was acquired using excitation through white diffused paper. Specific optical filters were used for fluorescence imaging. The spatial frequency patterns were projected using the DMD. For HSF-FLI, the time-resolved fluorescence signals were captured with a projected pattern at a phase shift of 2*π*/3 and 4*π*/3. Imaging settings, including emission power, micro-channel plate (MCP) voltage, camera exposure time and hardware binning ratio were adjusted based on the pre-formulated look-up table (LUT) of the ICCD accordingly [51].

#### 2.1.3 Lifetime Parameter Extraction

In the case of FRET, the FLI based parameters of interest are typically estimated through non-linear least square fitting (NLSF), described in equation (1). In this approach, the fluorescence decays are modeled by

\begin{equation*} \Gamma\left(t\right) = \mathrm{IRF}\left(t\right) * \left[ A_1 \mathrm{e}^{-t/\tau_1} + A_2 \mathrm{e}^{-t/\tau_2} \right]\end{equation*}

where the experimental decay, $\Gamma(t)$, is the combination of a bi-exponential model convolved with pixel-wise IRF(*t*). The FLI-based parameters of interest are the donor *τ*_1_, acceptor lifetimes *τ*_2_ and corresponding amplitude *A*_1_ and *A*_2_. The FRET percentage, *f*_1_, could thus be obtained through equation (2),

\begin{equation*} f_{1} = \frac{A_1 \left(1 - \exp\left(-\frac{T}{\tau_1}\right)\right)}{\sum_{i} \left(A_i \left(1 - \exp\left(-\frac{T}{\tau_i}\right)\right)\right)}\end{equation*}

where the amplitude weighted average lifetime of the two lifetime components, *τ*_a_, is used as the parameter to obtain the correct energy transfer efficiency (3),

\begin{align*} \tau_\textrm{a} = f{1}*\tau_1 +\left(1-f_1\right)*\tau_2\end{align*}

The systemic effects on the IRF and the fluorescence decay curve (FDC) have been studied for the system earlier [24, 51, 52]. However, the acquired fluorescence decays can originatie from different tissue depths that are not distinguishable directly from the measurements. Hence, the FLI-based parameter estimation may be affected by the heterogeneous nature of the biological tissue under investigation.

### 2.2 Spatial frequency domain imaging optical property extraction

Optical properties of the agar-based 20% intralipid phantoms are extracted through spatial frequency domain imaging (SFDI) using the MFLI system with a gate width of 1000 ps [24]. Three pre-made 8-bit sinusoidal Labview patterns at a spatial frequency of 0.1 $\textrm{mm}^{-1}$ with offset of 2*π*/3 were projected through DMD. A factory-characterized silicon phantom (INO Biomimic Phantoms, QC) with known optical properties, absorption coefficient *µ*_a_, and reduced scattering coefficient $\mu_\textrm{s}^{^{\prime}}$ (*µ*_a_ = 0.03 $\textrm{mm}^{-1}$, $\mu_\textrm{s}^{^{\prime}}$ = 1.15 $\textrm{mm}^{-1}$ at 700 nm, and *µ*_a_ = 0.03 $\textrm{mm}^{-1}$, $\mu_\textrm{s}^{^{\prime}}$ = 1.2 $\textrm{mm}^{-1}$ at 760 nm) was used to calibrate the optical properties of the tissue mimicking phantoms used for experiment. A total of 10 gates with 500 ps step were acquired for each illumination pattern using ND filter for both intralipid-based agar phantom and the calibration phantom under both 700 nm and 760 nm. Both phantoms were imaged at the same distance from the camera to avoid the need to perform height correction. Optical properties including *µ*_a_ and $\mu_\textrm{s}^{^{\prime}}$ were obtained through a multi-frequency LUT.

### 2.3 HSF-FLI MTF

HSF-FLI was derived from the optical sectioning through the deconvolution of the non-depth dependent FLI signal, $I_\textrm{DC}$, to derive the spatially modulated surface signal, $I_\textrm{AC}$, and non-modulated subsurface signal, $I_\textrm{sub}$. Fluorescence planar signal, $I_\textrm{DC}$, and surface scattered signal, $I_\textrm{AC}$, could also be demodulated based on the phase offset signals, $I_1,\text{ }I_2,\text{ and }I_3$, due to the constant phase shift of 2*π*/3 in equation (4),

\begin{equation*} \left\{ \begin{array}{l@{}l} I_\textrm{DC} = I_\textrm{AC} + I_\textrm{sub} = \left(I_1 + I_2 + I_3\right)/3 \\[3pt] I_\textrm{AC} = \frac{\sqrt{2}}{3} \sqrt{\left(I_1 - I_2\right)^2 + \left(I_2 - I_3\right)^2 + \left(I_3 - I_1\right)^2}. \end{array} \right.\end{equation*}

To quantify the efficiency of different spatial frequencies in HSF-FLI, MTF, was derived for fluorescence imaging from MTF in Spatial Frequency Domain, SFD, [53, 54] and used for this work in equation (5),

\begin{equation*} \left\{ \begin{array}{l@{}l} \textrm{MTF}\left(f_x\right) &amp; = M_\textrm{R}^{\left(f_x\right)} / M_\textrm{S}^{\left(f_x\right)} \\[3pt] M_\textrm{R}\left(f_x\right) &amp; = A_\textrm{R}^{\left(f_x\right)} / A_\textrm{R}^{\left(0\right)} = I_\textrm{AC}^{\left(f_x\right)} / I_\textrm{DC} \\[3pt] M_\textrm{S}\left(f_x\right) &amp; = A_\textrm{S}^{\left(f_x\right)} / A_\textrm{S}^{\left(0\right)} \end{array} \right.\end{equation*}

where $M_\textrm{R}$ is the modulation depth of the reflectance from the sample and is the fraction of reflected DC amplitude, $A_\textrm{R}^{(f_x)}$, and reflected AC amplitude, $A_\textrm{R}^{(0)}$, which are the same as $I_\textrm{AC}$ and $I_\textrm{DC}$ respectively. $M_\textrm{S}$ is the modulation depth of source fluence and is the fraction of DC source amplitude, $A_\textrm{S}^{(f_x)}$, and AC amplitude, $A_\textrm{S}^{(0)}$, from the source.

In HSF-FLI, the source fluence is modulated by patterns with offsets, and the reflectance is directly captured through the Gated-ICCD detector. In this case, $M_\textrm{S}$ could thus be calculated through equation (6),

\begin{equation*} M_{S}\left(f_x\right) = A_\textrm{p}^{\left(f_x\right)} / A_\textrm{p}^{\left(0\right)} = I_{P_\textrm{AC}}^{\left(f_x\right)} / I_{P_\textrm{DC}}\end{equation*}

where $A_\textrm{p}^{(0)}$ and $A_\textrm{p}^{(f_x)}$ and is the amplitude of the projected patterns with DC and AC components in equation (7) and $ I_{P_\textrm{DC}}$ and $ I_{P_\textrm{AC}}$ are pattern modulated source fluence of non-depth dependent and spatially modulate signal respectively,

\begin{equation*} \left\{ \begin{array}{l@{}l} I_{P_\textrm{DC}} = \left(I_\textrm{p1} + I_\textrm{p2} + I_\textrm{p3}\right)/3 \\[3pt] I_{P_\textrm{AC}} = \frac{\sqrt{2}}{3} \sqrt{\left(I_\textrm{p1} - I_\textrm{p2}\right)^2 + \left(I_\textrm{p2} - I_\textrm{p3}\right)^2 + \left(I_\textrm{p3} - I_\textrm{p1}\right)^2} \end{array} \right.\end{equation*}

which leads to the finalized MTF equation for HSF-FLI as equation (8)

\begin{equation*} \textrm{MTF}\left(f_x\right) = \frac{M_\textrm{R}\left(f_x\right)}{M_\textrm{S}\left(f_x\right)} = {\frac{I_\textrm{AC}^{\left(f_x\right)}}{I_\textrm{DC}} \cdot \dfrac{I_{P_\textrm{AC}}^{\left(f_x\right)}}{I_{P_\textrm{DC}}}}.\end{equation*}

### 2.4 MC eXtremeMCX simulation

The details of the MCX simulation workflow [55–57] used to model fluorescence emission/collection at the phantom surface are thoroughly described elsewhere [58]. Briefly, computational phantoms are designed to contain fluorescent inclusions with varying shapes and depths. Each inclusion can be assigned heterogeneous relative quantum yields and multiple fluorescence lifetimes, representing complex, multiplexed imaging scenarios. The generation of fluorescence data consists of two primary steps. First, forward light propagation at the excitation wavelength is simulated using experimentally derived structured illumination patterns to determine the temporal profiles of light fluence at each discretized element within the inclusions. These spatio-temporal excitation intensities serve as emission sources, incorporating fluorescence photophysics through local quantum yields and lifetime parameters. Next, a second MC simulation is performed to model the propagation of the emitted fluorescence photons from the excitation sites to the phantom surface. The resulting temporal fluorescence profiles are then convolved with the experimentally measured IRF, yielding a detailed representation of the fluorescence decay kinetics as observed on the surface.

### 2.5 Animal experiments

Two 4 weeks old athymic female nude mice (CrTac: NCr-Foxn1nu; Taconic Biosciences), were injected with 200 *µ*L of $10\times10^{6}$ HER2+ AU565 breast cancer cells (ATCC) mixed 1:1 with Cultrex BME (R$\&amp;$D Systems Inc., Minneapolis, MN, USA) into the right and left inguinal mammary fat pads. After 4 weeks of tumor growth, NIR labeled Meditope (MDT) TZM probes were injected retro-orbitally. 20 *µ*g of MDT-conjugated TZM-AF700 probe was administered retro-orbitally in the non-FRET mouse, and for the FRET mouse, 20 *µ*g MDT-TZM-AF700 (Donor)/40 *µ*g MDT-TZM-AF750 (Acceptor) FRET pair probes were injected through retro-orbital route with a difference of 2 h between donor-labeled and acceptor-labeled drug injections. Both the MDT-non-FRET and FRET pair probes were generously provided by Dr John C. Willams lab Department of Molecular Medicine, Beckman Research Institute of City of Hope, Duarte, CA. The HSF-FLI was performed at 48hr post injection in anesthetized (EZ-SA800 System, E–Z Anesthesia, USA) mice placed on a $37{\,}^{\circ}$C warming pad (Rodent WarmerX2, Stoelting, IL, USA) on the imaging stage. After euthanasia, the tissues including skin and fat above tumors were carefully removed to expose the tumor for *ex vivo* imaging. The tumors were kept within the mouse body to reduce the effects of changing fluorescence environments. All animal protocols were conducted with approval by the Institutional Animal Care and Use Committee at both Albany Medical College and Rensselaer Polytechnic Institute.

## 3 Results

**Figure 1:**
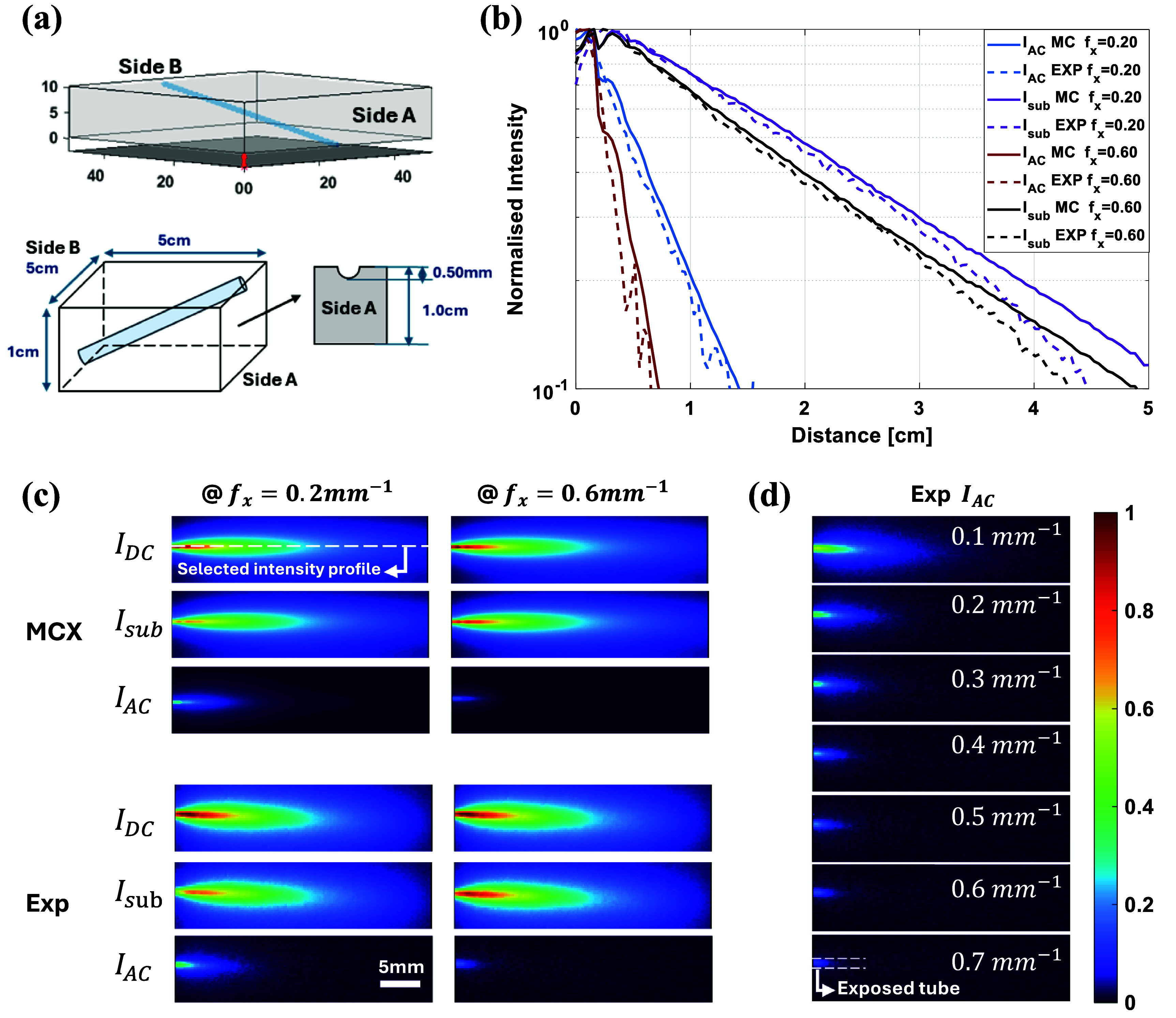
Comparison of fluorescence intensity profiles between MCX simulations and experiments, (a) the structure of MCX phantom on the top, and design of the experimental agar-based capillary phantom filled with 1 *µ*M AF700 on the bottom. (b) Normalized intensity distribution from side A to B at selected fx follow selected intensity profile in dotted line. (c) Normalized fluorescence planar signal, $I_\textrm{DC}$, and calibrated subsurface signal, $I_\textrm{AC}$, of a tilted tube at three different spatial frequencies, *fx* = 0.2, 0.4, 0.6 $\textrm{mm}^{-1}$. (d) Experimental $I_\textrm{AC}$ changing as spatial frequency increasing.

MCX simulations were performed on a tilted capillary phantom and directly compared with experimental measurements to assess whether the simulated datasets can be used to correlate spatial frequency with depth information in practical applications in figure 1. The bulk medium was assigned optical properties of $\mu_\textrm{a} = 0.002\ \mathrm{mm}^{-1}$ and $\mu_\textrm{s}^{^{\prime}} = 1\ \mathrm{mm}^{-1}$ to align with the SFDI calibrated experimental phantom optical properties, while the interior dye solution was set to $\mu_\textrm{a} = 0.04\ \mathrm{mm}^{-1}$ and $\mu_\textrm{s}^{^{\prime}} = 0.09\ \mathrm{mm}^{-1}$. The design and the selection of optical properties for both bulk medium and fluorescent dye could be found in previous MCX work [58]. The MCX simulation processes were described in the methods section and the detected fluorescence signal from the 2-step MCX simulation is shown in figure 1(a) top. For validation, the capillary phantom was filled with 1 *µ*M Alexa Fluor 700 in an intralipid scattering medium shown in figure 1(a) bottom. Data were acquired at spatial frequencies from 0.1 to 0.7 $\textrm{mm}^{-1}$ in 0.1 $\textrm{mm}^{-1}$ steps; results at 0.2 and 0.6 $\textrm{mm}^{-1}$ are shown here, with the full frequency series in supplementary figures S1 and S2.

To compare simulation and experiment, the profiles of $I_\textrm{AC}$ and $I_\textrm{sub}$ were extracted following the tube from side A to B and normalized to the peak value of each profile in figure 1(b), and the selected intensity profile was shown in the white dotted line in figure 1(c). A section of the tube was deliberately exposed on side A for surface reference; profiles not exceeding unity are displayed. Despite the noise in the experimental curves, both $I_\textrm{AC}$ and $I_\textrm{sub}$ agree closely between simulation and measurement at the selected frequencies and the corresponding values of *R*^2^ are included in table 1. Finally, after normalizing by $I_\textrm{DC}$, we compared $I_\textrm{DC}$, $I_\textrm{AC}$, and $I_\textrm{sub}$ across all frequencies: $I_\textrm{sub}$ amplitude increases with spatial frequency—indicating shallower sampling depths—whereas $I_\textrm{AC}$ captures deeper regions at low frequencies and shifts toward shallower depths as the frequency increases in figure 1(d).

**Table 1:** *R*^2^ of MXC simulated and Experimental intensity differences at spatial frequency range from 0.1 to 0.7 $\textrm{mm}^{-1}$ with 0.1 $\textrm{mm}^{-1}$ step size.

	Spatial frequency ($\mathrm{mm}^{-1}$)						
	0.10	0.20	0.30	0.40	0.50	0.60	0.70
$I_{\mathrm{AC}}$	0.993	0.988	0.975	0.953	0.970	0.957	0.926
*I*_sub_	0.972	0.982	0.988	0.987	0.989	0.990	0.989

Under high spatial-frequency conditions, accurate projection and imaging of structured patterns with sufficient resolution is critical for reliable analysis. Therefore, determining the maximum spatial frequency that can be experimentally achieved without loss of information is essential. Given the specifications of our gated ICCD (Picostar HR, LaVision GmbH, Bielefeld, Germany) and DMD (DLi 4110, Texas Instruments, TX, USA) imaging system, we estimated the system’s maximum usable spatial frequency to be 0.6 $\textrm{mm}^{-1}$. This limit was established by evaluating the determination coefficient (*R*^2^) for $I_\textrm{AC}$, which decreased from 0.957 to 0.926 as the spatial frequency increased from 0.6 to 0.7 $\textrm{mm}^{-1}$ (supplementary figure, S2). These results indicate that the accuracy of the projection of sinusoidal patterns degrades at spatial frequencies above 0.6 $\textrm{mm}^{-1}$.

**Figure 2:**
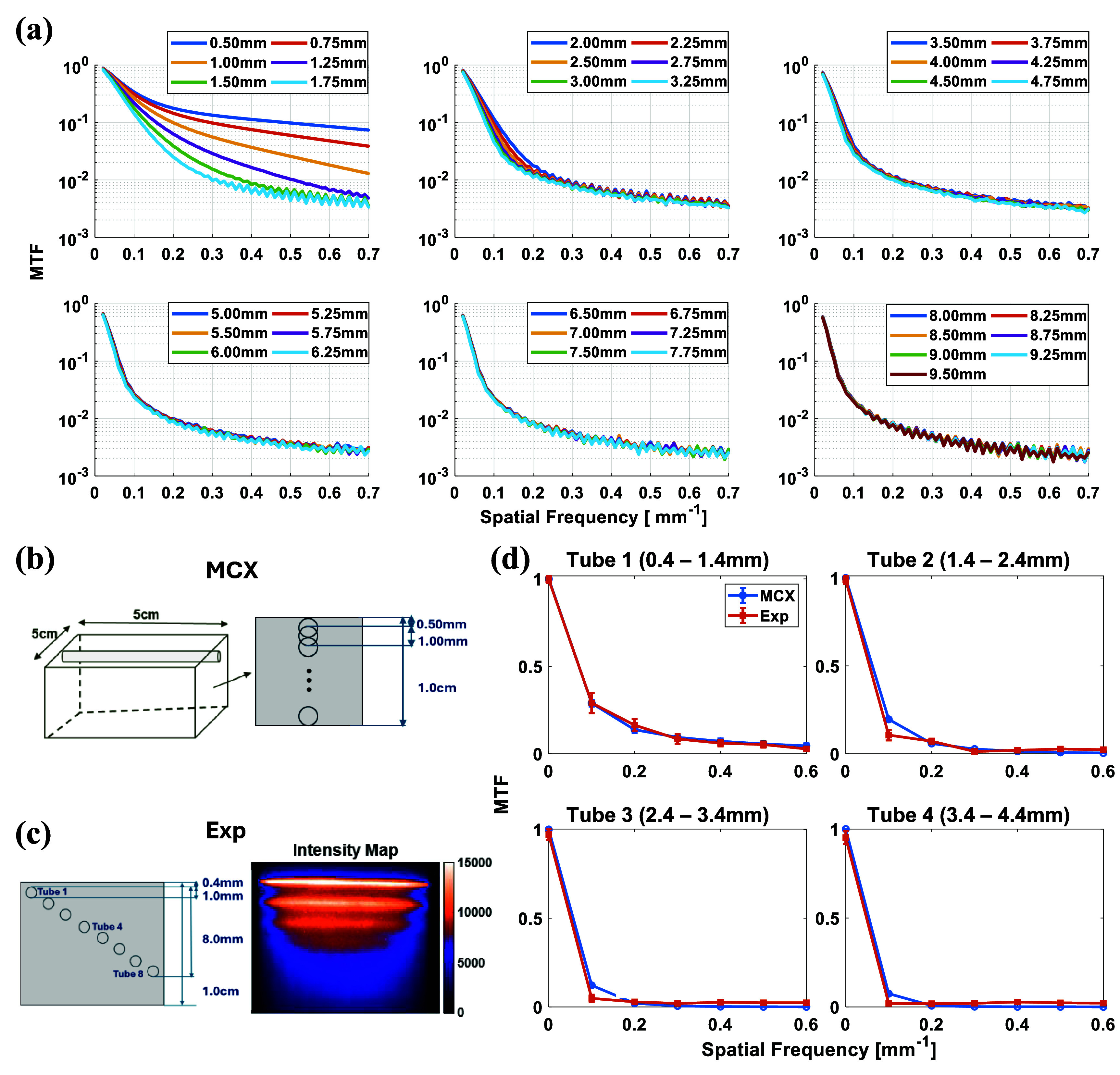
Variation of MTF with spatial frequencies, (a) depth-related MTF through MCX simulated fluorescence data, *f_x_* = 0.01 to 0.70 $\textrm{mm}^{-1}$ in steps of 0.01 $\textrm{mm}^{-1}$. (b) MCX phantom design with capillaries shifted downwards progressively in steps of 0.25 $\textrm{mm}$. (c) Experimental agar-based capillary phantom, $\mu_\textrm{s}^{^{\prime}}$ = 1.00 $\textrm{mm}^{-1}$ with 700 nm excitation wavelength through SFDI, designed with 8 1 $\textrm{mm}$ glass capillaries filled with 1 *µ*M AF700 sample. Tube 1 is approximately 0.4 $\textrm{mm}$ beneath the surface, and the corresponding intensity Map. (d) MTF comparison between first 4 tubes in experimental phantom and MCX at the same depth with *f_x_* = 0.0 to 0.60 $\textrm{mm}^{-1}$ in steps of 0.01 $\textrm{mm}^{-1}$.

With the validity of MCX simulations established for experimental conditions (in phantom), we quantified fluorescence-signal sensitivity variation with spatial frequency via the MTF defined in equation (8). Depth-resolved MTF profiles—spanning from 0.50 $\textrm{mm}$ to 9.50 $\textrm{mm}$ into the capillary—are presented in figure 2(a) and the simulation schematic is shown in figure 2(b). Spatial frequency was incremented in 0.01 $\textrm{mm}^{-1}$ steps. At the zero-frequency limit (*f_x_* = 0 $\textrm{mm}^{-1}$), $I_\textrm{AC}$ and $I_\textrm{DC}$ are identical, yielding an MTF of 1. As frequency increases, the MTF steadily declines across all depths, reflecting the reduced ability to resolve finer patterns at greater penetration. At selected spatial frequencies for experimental conditions, *f_x_* = 0.6 $\textrm{mm}^{-1}$, the MTF drops below 0.01, two orders of magnitude down, beyond depths of roughly 1.5 $\textrm{mm}$ to 1.75 $\textrm{mm}$, indicating that this frequency represents a practical resolution limit in depth. This trade-off between spatial resolution and penetration depth underscores the importance of selecting frequencies that balance pattern fidelity with sufficient sampling depth in MFLI experiments.

To validate the accuracy of the MCX generated MTF, the experimental phantom was designed to have 8 tubes each at 1 $\textrm{mm}$ depth incremented from the previous in total in an agar-based intra-lipid phantom with *µ*_a_ = 0.02 $\textrm{mm}^{-1}$ and $\mu_\textrm{s}^{^{\prime}}$ = 1 $\textrm{mm}^{-1}$ characterized under 700 nm excitation wavelength. The structure of the phantom is shown in figure 2(c). Each tube was filled with 1 *µ*M AF700, producing the fluorescence intensity map shown in figure 2(c) right. In the phantom experiment, only first four tubes were observable under white field condition, *f_x_* = 0 $\textrm{mm}^{-1}$, in the intensity map and they were further selected as the targets for comparing the MTF with MCX simulated MTF at similar heights shown in figure 2(d) at depths of 0.4 $\textrm{mm}$, 1.4 $\textrm{mm}$, 2.4 $\textrm{mm}$, and 3.4 m. Shallower tubes exhibited sharper boundaries due to low signal attenuation, while deeper tubes appeared progressively diffuse. At 0.4 $\textrm{mm}$ depth, simulated and experimental MTF curves aligned with an *R*^2^ of 0.98 across spatial frequencies from 0 to 0.6 $\textrm{mm}^{-1}$. Correlation coefficients decreased with depth (*R*^2^ = 0.95 at 1.4 $\textrm{mm}$, 0.91 at 2.4 $\textrm{mm}$, and 0.87 at 3.4 $\textrm{mm}$), reflecting increased scattering and signal attenuation. From the comparison between both the intensity profile shown in figure 1 and MTF of MCX and experimental results, the MCX simulation showed good correspondence as the experiential, and the MCX-derived MTFs accurately emulate real fluorescence-lifetime measurements and are suitable for direct application in experimental settings.

**Figure 3:**
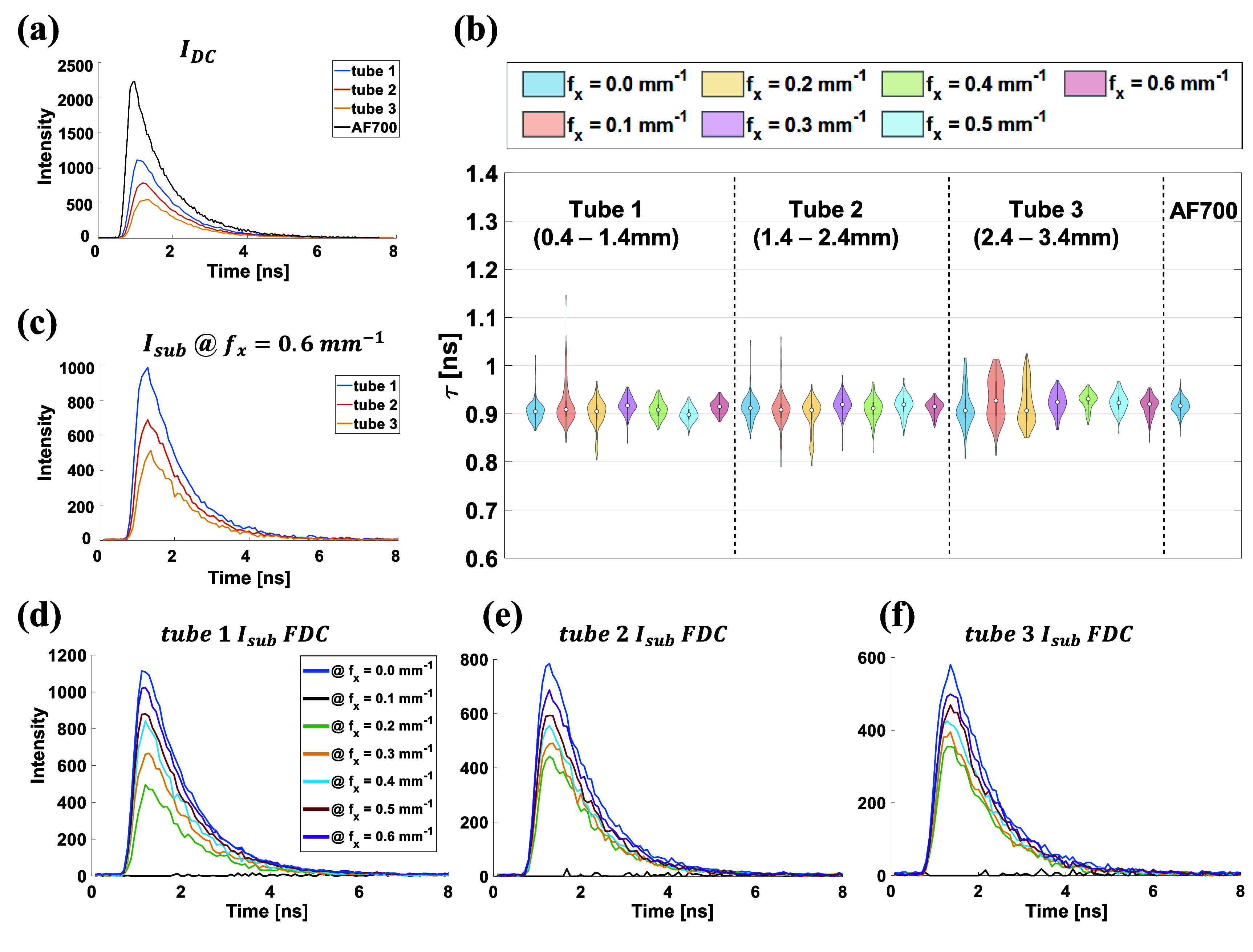
Time domain HSF-FLI of first three tube in figure 2, (a) fluorescence decay curve (FDC) of $I_\textrm{DC}$, for three tubes and the control fluorescence dye in wellplate, 1 *µ*M AF700. (b) Lifetime results from NLSF fitted $I_\textrm{sub}$ at different spatial frequencies of tube 1, 2, and 3, and wellplate AF700 control. (c) Reconstructed $I_\textrm{sub}$ at *f_x_* = 0.6 $\textrm{mm}^{-1}$ comparison among three tubes. Reconstructed $I_\textrm{sub}$ for *f_x_* = 0.10 to 0.60 $\textrm{mm}^{-1}$ in steps of 0.01 $\textrm{mm}^{-1}$ of (d) tube 1, (e) tube 2, (f) tube 3.

Following strong agreement between MTFs from MCX simulations and phantom measurements in continuous wave (CW) acquisition, the time-resolved signal using HSF illumination was captured for fluorescence lifetime estimation (referred to hereafter as HSF-FLI). HSF-FLI determined fluorescence lifetime after surface-scattering contributions $I_\textrm{AC}$ were removed. We limit this analysis to the three shallowest tubes of the capillary in phantom depicted in figure 2. Figure 3(a) presents fluorescence-decay curves (FDCs) of the depth-independent signal $I_\textrm{DC}$ from these tubes alongside a 1 *µ*M AF700 reference acquired under scattering-free conditions in a well plate. All images of phantom and well-plate were captured using identical instrument settings to avoid lifetime-analysis bias from varying noise conditions. In the absence of scattering, the AF700 well plate signal peaked at approximately 2300 camera counts. Under scattering in tube 1, the peak intensity decreased by roughly 50 $\%$, with further reductions observed at greater tube depths.

Following removal of the surface signal, $I_\textrm{sub}$ at each spatial frequency underwent NLSF analysis for each of the three capillary tubes; the resulting lifetimes are displayed in figure 3(b). Corresponding intensity, lifetime, and *R*^2^ are included in table S1, while representative fits of each tube at each spatial frequency are shown in figure S3. The number of pixels included in the fitting are provided in table S4. Standard deviation of the fitted lifetimes declined as the spatial frequency increased, while the mean lifetime remained near 0.9 ns across all tubes. Deeper tubes exhibited larger overall lifetime variability, correlating with reduced fluorescence decay signal quality shown in figures 3(d)–(f). Representative fluorescence-decay curves for $I_\textrm{sub}$ at 0.6 $\textrm{mm}^{-1}$ shown in figure 3(c) reveal depth-dependent noise artifacts that drive NLSF uncertainty, as reflected in the standard deviations plotted in figure 3(b). In all cases, the NLSF provided excellent fits, with *R*^2^ consistently above 0.996. Notably, at 0.6 $\textrm{mm}^{-1}$, *R*^2^ values were equal to or slightly higher than those at 0 $\textrm{mm}^{-1}$, indicating that the removal of the surface signal yields decay profiles more consistent with a mono-exponential model dominated by the embedded fluorophore.

At 0.4 $\textrm{mm}$ depth (tube 1), the capillary’s lifetime distribution narrows and aligns with that of the AF700 control at 0.4 $\textrm{mm}^{-1}$, reaching its tightest spread at 0.6 $\textrm{mm}^{-1}$. Tube 1 also maintained the highest overall intensity across spatial frequencies (table S1), supporting the stable lifetime fits. At 1.4 $\textrm{mm}$ depth (tube 2), the lifetime standard deviation grows across all spatial frequencies compared to tube 1 but remains on par with the well plate sample. Here, the intensity decreased more rapidly with frequency compared to tube 1, while *R*^2^ values remained consistently high ($\unicode{x2A7E}$0.997), confirming that the mono-exponential model still provided robust fits despite a weaker signal. In the deepest tube (2.4 $\textrm{mm}$), the standard deviation again decreases at higher spatial frequencies, although small shifts in the mean lifetime likely reflect the substantially lower signal intensity. As expected, tube 3 exhibited the lowest intensity values overall, but its *R*^2^ values stayed above 0.996 at every frequency, indicating that even at reduced photon counts, the model accurately described the decay behavior. In this case, even with the subtraction of the surface signal, the related subsurface signal FDCs could still be used in the NLSF for lifetime analysis shown in figures 3(d), (e) and (f). As the depth of the fluorescence signal increases, the subsurface FDCs exhibit more fluctuations as the spatial frequencies of the illumination pattern decrease. The smaller distribution at depths of 0.4 $\textrm{mm}$ and 1.4 $\textrm{mm}$ at 0.6 $\textrm{mm}^{-1}$ compared to the control is related to the size of the samples.

With more pixels existing in the wellplate sample, 1071, compared to a single tube, 154, more variation could be introduced by both imaging noise and fitting variations [51]. For the AF700 control, which contained pure dye without scattering medium, the intensity was substantially higher and the lifetime distribution remained tightly centered around 0.9 ns with *R*^2^ values exceeding 0.998. In contrast, the capillary tubes embedded in the scattering phantom exhibited lower intensity and broader distributions due to photon diffusion and reduced photon counts. Importantly, applying HSF patterns in HSF-FLI effectively suppressed superficial scattering and recovered subsurface decays that converged toward the true dye lifetime, demonstrating the method’s ability to correct for scattering effects and improve depth-specific accuracy.

**Figure 4:**
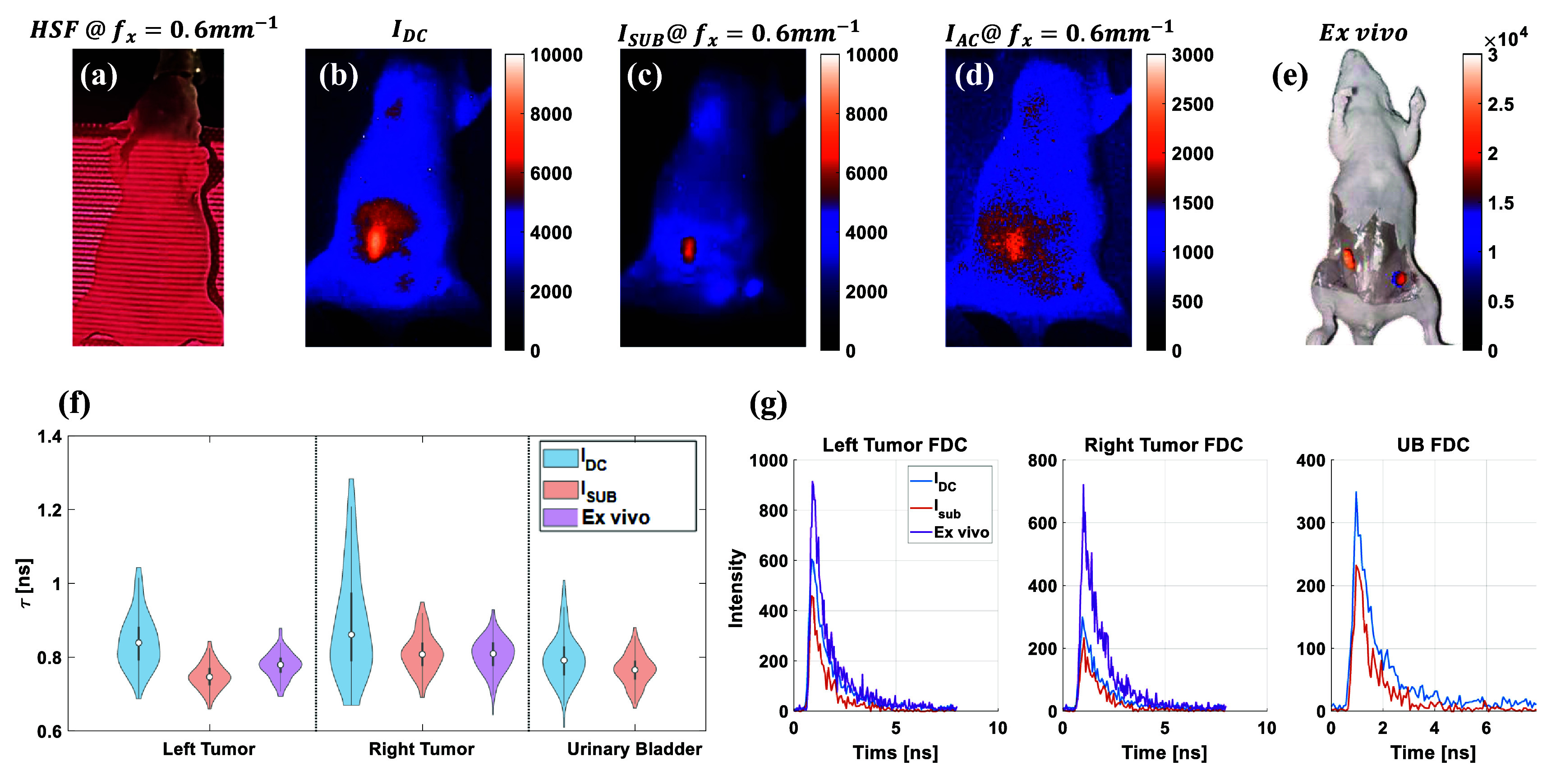
Time domain HSF-FLI of *in vivo* sample injected with MDT-TZM-AF700, (a) HSF projection of *in vivo* sample at *f_x_* = 0.6 $\textrm{mm}^{-1}$, Fluorescence intensity map of *in vivo* sample at fx = 0.6 $\textrm{mm}^{-1}$ of (b)$I_\textrm{DC}$, (c) $I_\textrm{sub}$, (d) $I_\textrm{AC}$. (e) Fluorescence intensity map of skin opened *ex vivo* sample, tissue above tumors removed after euthanasia, skin removed *ex vivo* mouse. (f) Lifetime comparison of left tumor, right tumor, and urinary bladder from NLSF fitting of $I_\textrm{DC}$, $I_\textrm{sub}$, and *ex vivo* skin opened mouse FDCs. (g) FDCs used for NLSF fitting.

**Figure 5:**
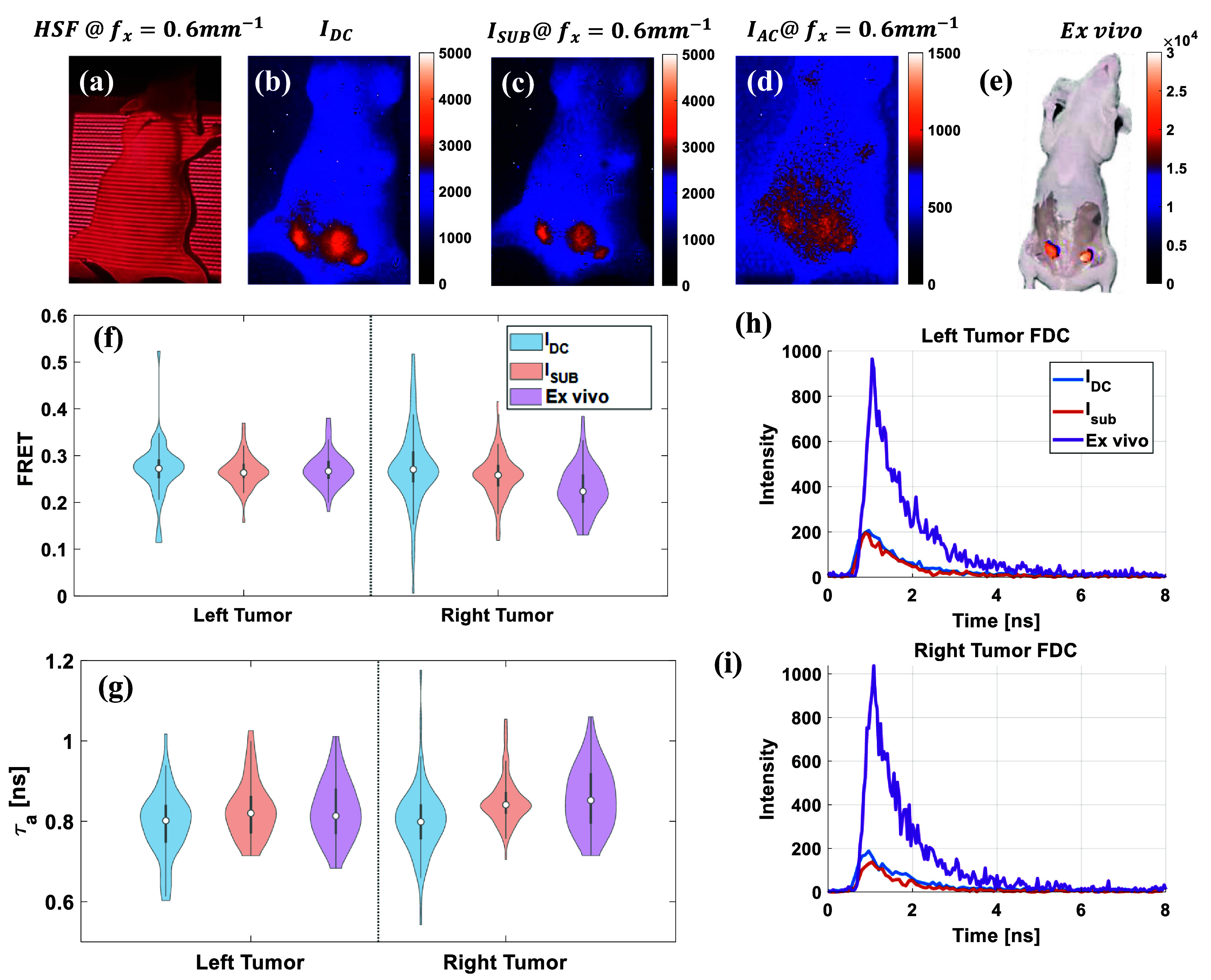
Time domain HSF-FLI of *in vivo* sample injected with MDT-TZM-AF700/MDT-TZM-AF750 FRET pair, (a) HSF projection of *in vivo* sample at *f_x_* = 0.6 $\textrm{mm}^{-1}$, fluorescence intensity map of *in vivo* sample at fx = 0.6 $\textrm{mm}^{-1}$ of (b)$I_\textrm{DC}$, (c) $I_\textrm{sub}$, (d) $I_\textrm{AC}$. (e) Fluorescence intensity map of skin opened *ex vivo* sample, tissue above tumors removed after euthanasia. Comparison of left tumor and right tumor, and urinary bladder from NLSF fitting of $I_\textrm{DC}$, $I_\textrm{sub}$, and *ex vivo* skin opened mouse FDCs for (f)FRET, and (g)Amplitude weighted mean lifetime, *τ*_a_, (h) FDCs used for NLSF fitting.

The HSF-FLI was further applied to non-FRET and FRET fluorescence probe labeled-mouse models bearing AU565 tumor xenografts. *In vivo* and *ex vivo* imaging was performed 48hr post-injection of NHS-conjugated MDT-TZM-AF700 non-FRET or MDT-TZM-AF700/MDT-TZM-AF750 FRET pair probes shown in figures 4 and 5. Traditional NHS-ester dye labeling leads to random, non-specific conjugation that compromises reproducibility, functional integrity, and quantitative accuracy [59–61]. To overcome these limitations, we have used MDT-TZM, an engineered antibody allowing site-specific labeling via a 12-amino acid cyclic peptide binding site in the Fab region [60, 62]. This modular system enables consistent dye conjugation without affecting HER2 binding or pharmacokinetics, improving imaging reliability, delivery, and therapeutic potential [62]. Therefore, in the present study we have used MDT-TZM conjugated with donor and acceptor NIR dyes as FRET pair probes to enable precise, reproducible, and quantitative imaging of HER2-positive tumors.

Based on the reported female mouse skin thicknesses of 400 to 500 *µ*m [63], a spatial frequency of 0.6 $\textrm{mm}^{-1}$ was chosen, and the structured-light pattern on the *in vivo* sample is shown in figure 4(a). In the wide-field, non-depth-resolved fluorescence image $I_\textrm{DC}$, tumor boundaries appear indistinct, and high signal from the gut overlaps with the left tumor shown in 4(b). Excess fluorescence from non-tumor regions was eliminated by HSF–FLI, yielding clear delineation of both tumors and the urinary bladder once the surface signal $I_\textrm{AC}$ is removed in 4(c) and (d). Upon removal of tissue above the tumor region, including skin and fat, the signal intensity increases approximately threefold in 4(e), creating a scattering-free reference. Lifetime values for tumors and bladder for non-depth dependent illumination ($I_\textrm{DC}$), subsurface signal from 0.6 $\textrm{mm}^{-1}$ spatial frequency pattern illumination, and *ex vivo* white field imaging are shown in the violin plots in figure 4(f). The number of pixels used for each organ are included in supplementary table S4. Under scattering conditions $I_\textrm{DC}$, lifetime distributions across all three regions of interest (ROIs) exhibit broad variability; in contrast, both surface-signal-subtracted $I_\textrm{sub}$ and *ex vivo* measurements show similar distributions and standard deviations. Corresponding intensity, lifetime, and *R*^2^ values are summarized in table S2, and representative decay fits are shown in figure S4. For the left tumor, intensity decreased from $7784 \pm 593$ cts, camera counts, to $4610 \pm 506$ cts when moving from $I_\textrm{DC}$ to $I_\textrm{sub}$, while the mean lifetime changed from $0.83 \pm 0.07$ ns to $0.75 \pm 0.04$ ns, consistent with the *ex vivo* value of $0.78 \pm 0.03$ ns. Similarly, the right tumor showed a similar trend where the intensity dropped from $4363 \pm 416$ cts to $2359 \pm 314$ cts, the lifetime stabilized near $0.81 \pm 0.05$ ns and *R*^2^ improved from 0.980 to 0.986 with relative low photon counts. In the urinary bladder, lifetime values stayed unchanged, $0.78 \pm 0.06$ ns to $0.77 \pm 0.04$ ns, but *R*^2^ increased slightly from 0.981 to 0.984, demonstrating that HSF–FLI maintained fit quality while reducing intensity from $4812 \pm 225$ cts to $2810 \pm 182$ cts. The absence of a bladder signal in *ex vivo* imaging is attributed to post-mortem urine expulsion and thus was excluded from lifetime analysis. High MCP voltage imaging settings in both *in vivo* and *ex vivo* experiments introduce noise into FDCs in figure 4(g), although peak intensities remain comparable to phantom and fluorophore measurements in figure 3. Despite these limitations, HSF–FLI reliably enhances ROI boundary contrast and suppresses superficial scattering, thereby improving fluorescence-lifetime specificity and precision.

HSF–FLI was applied to the FRET mouse bearing staggered injections (2 hr interval between donor and acceptor injection) [59] of MDT-TZM-AF700 and MDT-TZM-AF750, with the structured-light pattern, 0.6 $\textrm{mm}^{-1}$, generated by the DMD shown in figure 5(a). In the wide-field, non-depth-resolved intensity map $I_\textrm{DC}$, fluorescence signal from the lower abdomen spreads across tumor and bladder ROIs, rendering their boundaries indistinct; additional signal spillover from the liver and upper-left flank is also apparent in figure 5(b). Consistent with table S3, depth independent $I_\textrm{DC}$ intensity was $3188 \pm 252$ cts in the left tumor and $2809 \pm 276$ cts in the right tumor, of which only $2390 \pm 216$ cts and $1973 \pm 235$ cts, respectively was retained in $I_\textrm{sub}$, reflecting removal of superficial contributions. Under high-spatial-frequency illumination, undesired superficial contributions are effectively suppressed, producing separable ROIs in figures 5(c) and (d). Following euthanasia and skin and partial superficial tissue removal, the intensity map shown in figure 5(e) exhibits a sixfold increase in peak signal relative to the *in vivo* condition.

FRET fractions were quantified via bi-exponential NLSF according to equation (2), and the resulting standard deviations for both tumors decreased with high-frequency illumination and tissue removal in figure 5(f). For the left tumor, the FRET fraction shifted from $0.27 \pm 0.06$ under *DC* illumination to $0.26 \pm 0.04$ with HSF–FLI, closely matching the *ex vivo* reference of $0.27 \pm 0.03$. A similar convergence was observed in the right tumor ($0.27 \pm 0.08$ to $0.26 \pm 0.05$), approaching the *ex vivo* value of $0.23 \pm 0.05$. Amplitude-weighted lifetimes, *τ*_a_, calculated as equation (3), which reflect donor quenching, followed the same trend as shown in figure 5(g). The left tumor *τ*_a_ narrowed from $0.80 \pm 0.09$ ns to $0.82 \pm 0.06$ ns, aligning with the *ex vivo* value of $0.82 \pm 0.07$ ns, while the right tumor shifted from $0.81 \pm 0.08$ ns to $0.86 \pm 0.07$ ns, in agreement with the *ex vivo* reference of $0.86 \pm 0.07$ ns. Importantly, *R*^2^ values increased under HSF–FLI (e.g. 0.973 to 0.976 in the left tumor, 0.969 to 0.978 in the right), indicating more stable fitting. These results demonstrate that HSF imaging reduces scattering artifacts and yields FRET fractions and lifetimes that more closely approximate *ex vivo* measurements, underscoring its utility for accurate *in vivo* quantification. Fluorescence-decay curves used for FRET fitting are presented in figures 5(h) and (i) and representative decay fits for selected *in vivo* and *ex vivo* organs are shown in figure S5; although overall *in vivo* intensities decrease due to donor quenching, decay profiles remain noisy owing to the high-gain imaging settings.

## 4 Discussion

The HSF-FLI framework leverages structured illumination to isolate subsurface fluorescence signals and thereby improve the precision and specificity of lifetime measurements in scattering media. By deriving an HSF-FLI MTF through both MCX simulations and experimental phantoms, a strong correlation was observed between simulated and measured data, demonstrated by $R^2\unicode{x2A7E}$ 0.98 at 0.4 $\textrm{mm}$ to $R^2\unicode{x2A7E}$ 0.87 for depths extending to 3.4 $\textrm{mm}$. This agreement confirms the robustness of MCX data simulation pipeline, which can accurately estimate the depth-dependent attenuation of high-frequency structured light in high scattering media, and thus provides an essential quantitative basis for selecting spatial frequencies that suppress surface-scattered light while preserving subsurface fluorescence signals in MFLI.

The time-resolved fluorescence signals captured through HSF illumination translate CW structured-illumination optical sectioning into FLI optical sectioning. These signals can be exploited to subtract the depth-dependent time-resolved signal attenuation of fluorescence inclusion. By selectively filtering out surface-scattered fluorescence, time-resolved HSF-FLI isolates deep-tissue signals with exceptional specificity. This targeted removal of superficial contributions significantly reduces the lifetime estimation variation and uncovers subtle subsurface dynamics, all while preserving mean lifetime values. Moreover, the close concordance between lifetimes extracted from the subsurface component $I_\textrm{sub}$ under HSF-FLI and those measured under scattering-free conditions validates the technique’s ability to recover accurate lifetimes in high scattering media, establishing a robust foundation for its application in complex biological environments.

Depth-independent fluorescence $I_\textrm{DC}$ was de-convoluted into surface-scattered $I_\textrm{AC}$ and subsurface $I_\textrm{sub}$ components via phase-shifted sinusoidal illumination from DMD. Nonlinear least-squares fitting of $I_\textrm{sub}$ revealed a systematic reduction in lifetime variability as spatial frequency increased, the standard deviation of fitted lifetimes declined by nearly 50$\%$ at 0.6 $\textrm{mm}^{-1}$, while the mean lifetimes remained within ${\sim}5$% of scattering-free references. These improvements were most pronounced in the shallowest phantom tube (0.4 $\textrm{mm}$ depth) but remained significant even at depths of 4.4 $\textrm{mm}$, where lifetime precision under wide-field illumination was otherwise compromised by severe photon scattering. Comparison with AF700 controls confirmed that HSF-FLI lifetimes converged toward the pure-dye reference, indicating that scattering predominantly broadened distributions rather than shifting mean values.

*In vivo* and *ex vivo* validation of this method in mouse models further confirmed its efficacy. This was shown (in figures 4 and 5) by consistently suppressing superficial fluorescence contributions and improving the delineation of tumors and the urinary bladder, both when a single fluorescence probe and when FRET-pair probes conjugated with a tumor-targeted drug were injected. In non-FRET experiments, lifetime distributions from $I_\textrm{sub}$ closely matched *ex vivo* measurements after skin removal, confirming that the HSF-FLI method can effectively negate the confounding effects of tissue scattering. Although *R*^2^ values *in vivo* (0.97 & 0.99) were slightly lower than those achieved in phantom tubes ($\unicode{x2A7E}$0.996), structured illumination consistently improved the fit quality relative to wide-field imaging. More importantly, $I_\textrm{sub}$ lifetimes in tumors (0.75 & 0.81 ns) converged toward *ex vivo* values (0.78 & 0.80 ns), demonstrating that HSF imaging suppresses scattering-induced distortions without introducing significant bias into the mean values.

For FRET experiments, HSF-FLI not only reduced the spread of fitted lifetimes and FRET fractions but also shifted both metrics closer to scattering-free references. In the right tumor, for example, FRET efficiency narrowed from $0.27 \pm 0.08$ under $I_\textrm{DC}$ to $0.26 \pm 0.05$ under $I_\textrm{sub}$, approaching the *ex vivo* value of $0.23 \pm 0.05$. Similarly, amplitude-weighted lifetimes converged from 0.81 ± 0.08 ns ($I_\textrm{DC}$) to 0.86 ± 0.07 ns ($I_\textrm{sub}$), matching the *ex vivo* measurement of 0.86 ± 0.07 ns. The consistent convergence toward *ex vivo* values underscores that scattering primarily degrades precision rather than producing large systematic bias. By recovering lifetimes and FRET fractions that align more closely with scattering-free conditions, HSF-FLI demonstrates clear utility for quantitative FRET imaging *in vivo*. HSF illumination HSF-FLI effectively reduced the standard deviation of calculated FRET efficiencies in tumors during FRET studies, outperforming wide-field non-depth resolved $I_\textrm{DC}$ data despite considerable system noise contributions. Together, these results demonstrate that HSF-FLI enhances both the precision and biological accuracy of *in vivo* fluorescence lifetime and FRET quantification, yielding measurements that more accurately represent the underlying molecular interactions.

HSF-FLI offers a significant and unprecedented advantage: to our knowledge, it is the first method to enhance fluorescence lifetime sensitivity in deep tissue using structured illumination alone. This eliminates the need for invasive procedures for sample preparation, use of optical clearing agents like tartrazine, or specific hardware modifications. Moreover, the ease of implementation makes HSF-FLI compatible with existing MFLI systems without any additional optics, dyes, or calibration procedures. Altogether, HSF-FLI delivers a self-contained solution for quantitative, high-fidelity imaging of subsurface fluorescence signals, opening the door to broader adoption in both preclinical research and clinical diagnostics.

When considered in the broader context of FLI techniques shown in table S5, HSF-FLI occupies an intermediate but unique position. Wide-field fluorescence lifetime imaging microscopy (FLIM) provides unmatched subcellular resolution, ∼0.2 *µ*m lateral, ∼0.6 *µ*m axial, but is restricted to superficial depths, ∼300 *µ*m, and requires long acquisition times of several hours per dataset, increasing the difficulty for *in vivo* or macroscopic applications. Conventional MFLI, on the other hand, achieves far greater probe penetration, ∼5 $\textrm{mm}$, with rapid acquisition, ∼2–3 min per dataset with lateral resolution of 1 $\textrm{mm}$, but suffers from inseparable depth information from integrating both surface and subsurface contributions. By contrast, HSF-FLI, *f_x_* = 0.6 $\textrm{mm}^{-1}$, obtained smaller lateral resolution, ∼0.83 $\textrm{mm}$, while providing depth selectivity to ∼1.75 $\textrm{mm}$ within a total acquisition time of only ∼6 min. Although the maximum probing depth of ∼3.4 $\textrm{mm}$ is lower than that of MFLI due to frequency-dependent attenuation, the ability to separate superficial from subsurface signals substantially enhances the biological interpretability particularly for drug delivery and FRET imaging.

Despite these promising findings, several limitations merit consideration. First, the practical maximum spatial frequency in the current ICCD–DMD system is constrained to approximately 0.6 $\textrm{mm}^{-1}$; beyond this threshold, the coefficient of determination for $I_\textrm{AC}$ drops from 0.957 to 0.926, indicating substantial pattern distortion. For our application in the macroscopic domain, the 0.6 $\textrm{mm}^{-1}$ structure pattern is the most suitable given the depth of the *in vivo* tumors (400–500 *µ*m). However, this limit can be extended by decreasing the detector field-of-view by adapting the ICCD camera with a higher magnification lens. This would allow a system to be adaptable to multiple applications with differing fields-of-view.

In addition, the imaging acquisition process is longer compared to traditional wide-field FLI due to the number of image acquisition sets required (2 spatial frequencies (0 & 0.6 $\textrm{mm}^{-1}$) and 3 phases for each of the selected spatial frequencies versus one acquisition at the same number of gates for traditional wide-field imaging). Future hardware improvements, such as faster gating electronics or adaptive optics, could improve the acquisition speed. Additionally, higher spatial frequencies inherently reduce photon throughput, leading to lower signal-to-noise ratios at greater depths. Mitigation of these trade-offs may involve optimizing illumination power, detector settings, or complementary photon-collection strategies. Overall, HSF-FLI provides a robust, experimentally validated pathway to mitigate tissue-scattering artifacts in MFLI, enabling more reliable *in vivo* molecular investigations.

Future extensions of the HSF-FLI paradigm include first combining multiple spatial frequencies in a single acquisition to reconstruct depth-resolved lifetime 3D maps. Moreover, translating HSF-FLI into the short-wave infrared window may offer deeper penetration and lower background auto-fluorescence, particularly valuable for clinical intra-operative imaging. Prospective applications in guided drug-delivery and receptor-ligand engagement studies, particularly those employing FRET-based reporters, stand to benefit from the improved quantitative accuracy and spatial specificity demonstrated here.

## 5 Conclusion

The HSF-FLI framework enables depth-selective macroscopic fluorescence lifetime measurements by leveraging structured illumination patterns. Both MCX simulations and phantom experiments confirm its ability to suppress surface fluorescence and isolate subsurface signals, significantly improving lifetime precision. Additionally, *in vivo* and *ex vivo* studies show sharply defined regions of interest and enhanced accuracy in FRET assays, even under high system noise conditions. While very high spatial frequencies can reduce pattern fidelity and photon throughput, optimized illumination and detection can overcome these challenges. Ultimately, HSF-FLI provides a robust framework for high-precision molecular imaging in living tissues.

## Data Availability

The data that support the findings of this study are openly available at the following URL/DOI: https://doi.org/10.6084/m9.figshare.29549903.v1. Supplementary Information available at https://doi.org/10.1088/2515-7647/ae0aa1/data1.
